# Long-term efficacy of left bundle branch pacing and biventricular pacing in patients with heart failure complicated with left bundle branch block

**DOI:** 10.3389/fcvm.2024.1363020

**Published:** 2024-02-29

**Authors:** Jia Li, Hongwei Yi, Jun Han, Hongwei Han, Xi Su

**Affiliations:** Department of Cardiology, Wuhan Asian Heart Hospital, Wuhan, Hubei, China

**Keywords:** left bundle branch pacing, biventricular pacing, cardiac resynchronization therapy (CRT), heart failure, left bundle branch block (LBBB)

## Abstract

**Background:**

Left bundle branch pacing (LBBP) can physiologically correct complete left bundle branch block (CLBBB), and has become the best alternative to biventricular pacing (BiVP).

**Objective:**

To compare the efficacy of LBBP and BiVP in patients with heart failure (HF) complicated with CLBBB.

**Methods:**

This was a single-center retrospective study. Patients with HF complicated with CLBBB who underwent successful cardiac resynchronization therapy (CRT) in Wuhan Asian Heart Hospital from June 2018 to June 2023 were enrolled and divided into LBBP group and BiVP group according to the pacing method. The primary endpoints were the absolute increase of left ventricular ejection fraction (LVEF), left ventricular end-diastolic diameter (LVEDD), and echocardiographic response rate. Secondary endpoints were all-cause mortality, heart failure hospitalization (HFH), NT-proBNP, paced QRS duration, pacing threshold, and procedural duration.

**Results:**

A total of 120 patients were enrolled in this study, including 60 patients in LBBP group and 60 patients in BiVP group. The median follow-up time was 37 ± 19 months. Compared with BiVP group, LBBP group had a more significant increase in absolute LVEF (ΔLVEF) (14.8 ± 9.9% vs. 10.7 ± 9.0%, *P *= 0.02), a more significant reduction in LVEDD (56.9 ± 10.9 mm vs. 61.1 ± 10.8 mm, *P *= 0.03), and a higher echocardiographic super response rate (65% vs. 45%, *P *= 0.02). There were no significant differences in all-cause mortality (1.7% vs. 10.0%, *P *= 0.11) and HFH (6.7% vs. 13.3%, *P *= 0.22). In terms of paced QRS duration (128.7 ± 14.1 ms vs. 137.5 ± 16.5 ms, *P *= 0.002), pacing threshold (0.72 ± 0.21 V/0.4 ms vs. 1.39 ± 0.51 V/0.4 ms, *P *< 0.001), procedural duration (134.1 ± 32.2 min vs. 147.7 ± 39.4 min, *P *= 0.04), the LBBP group was superior to the BiVP group.

**Conclusion:**

In nonischemic cardiomyopathy (NICM) patients with HF combined with CLBBB and LVEF ≤ 35%, LBBP is better than BiVP.

## 1 Introduction

Traditional biventricular pacing (BiVP) has been shown to reduce HFH and all-cause mortality in chronic HF patients with CLBBB and LVEF ≤ 35% (1). However, more than 30% of patients have no response to CRT (2–4). The incidence of adverse events such as phrenic nerve stimulation, threshold elevation and lead dislodgement in left ventricular coronary sinus(CS) lead can reach 10%∼30%. Compared with BiVP, His- bundle pacing (HBP), as the most physiological pacing method, can better achieve cardiac resynchronization and improve cardiac function, but its development is limited by its difficult implantation technology, high threshold, low perception, early battery exhaustion. In 2017, Huang et al. (5) reported a case of LBBB combined with HF patients with failed left ventricular lead implantation in CS for the first time, and the cardiac function was significantly improved after LBBP treatment. Since then, several studies have confirmed that LBBP has become an effective physiological pacing mode, which has the advantages of easier to cross the block area, low threshold and high perception compared with HBP. Recently, small randomized controlled trials have demonstrated that LBBP has better short-term efficacy than BiVP in nonischemic cardiomyopathy (NICM) combined with CLBBB (6). At present, there is a lack of long-term follow-up data on HF patients with CLBBB.

## 2 Methods

### 2.1 Research design

This was a retrospective, single-center, case-control study designed to evaluate the efficacy and differences between LBBP and BiVP in HF patients with CLBBB. The study included 120 patients with HF combined with CLBBB, LVEF ≤ 35%, New York Heart Association (NYHA) II-IV functional class despite optimal medical therapy and successfully receiving CRT-P/CRT-D for the first time in Wuhan Asia Heart Hospital from June 2018 to June 2023. According to pacing mode, 60 cases were in LBBP group and 60 cases in BiVP group. Patients with right bundle branch block (RBBB), intraventricular conduction block(IVCB), persistent atrial fibrillation, preexisting pacemaker implantation, left ventricular epicardial pacing, age < 18 years, and incomplete 6-month follow-up were excluded. All patients signed an informed consent before procedure, along with a detailed description of how strongly the guidelines recommended the two pacing methods. According to the experience and preference of the surgeon, some patients prefer LBBP, others prefer BiVP, and if unsuccessful, change to another way. This study follows the guidelines of the Declaration of Helsinki (revised in 2013). The echocardiographic response rates, defined as LVEF improved ≥5%, respectively, and super response rate, defined as both LVEF improvement ≥15% or LVEF ≥ 45%.

### 2.2 Interventional operation

#### 2.2.1 LBBP

The thickness of the ventricular septum was evaluated by echocardio-graphic before surgery, and the insertion depth of the lead was estimated. The Medtronic 3830-69 active fixation lead was implanted with the help of C315 HIS sheath tube (Medtronic, USA) via the left axillary vein route. The His bundle was mapped using 3830 electrodes or the apex of the tricuspid septum was shown by tricuspid ring imaging to estimate the His bundle position, and then the image was used as a marker to implant the left bundle branch pacemaker. In the right anterior oblique position of 30°, the line between His and the apex of the heart at 1 cm∼1.5 cm of the distal part of the bundle are selected as the initial position of LBBP. The pacing usually presents a “W” type QS wave on the *V*_1_ lead, and the sheath tube is rotated counterclockwise to keep the lead end perpendicular to the ventricular septum. When the lead is rotated from the right ventricular septum to the left ventricular septum, it can be found that: (1) During pacing, the collapse at the bottom of the QRS wave on the *V*_1_ lead will gradually move backward to the end of the QRS wave until the r wave at the end of the QRS wave appears. If the R wave continues to turn deeply, the *R* wave amplitude will increase, and the pacing pattern will be QR type, that is, the pacing pattern will change from LBBB to RBBB. (2) In high and low output pacing, pacing stimulus to left ventricular activation time (Stim-LVAT), that is, the time to the *R*-wave peak of the pacer nail to the *V*_4–6_ lead, was kept at the shortest and constant, indicating the capture of the left bundle branch. (3) Selective LBBP showed a separation between pacing stimulus and *V*-wave, and pacing ECG showed a typical RBBB. Non-selective LBBP appeared when the output voltage increased, and there was no separation between pacing stimulus and *V*-wave. At left anterior oblique 45°, the vertical insertion and the insertion depth of the lead were determined by sheath angiography. The intrathecal tube was withdrawn to the atrium, and the lead movement was observed. If the measured parameters were satisfactory and constant (≤1.0 V/0.4 ms) and the pacing pattern and intracavitary electrogram did not change, the auxiliary sheath was dissected. If LBBP was not successful, the BiVP was replaced. In patients with CRT-D or CRT-P, right ventricular leads (including defibrillation leads) were implanted in the right ventricular apex. The atrial leads (3830) were implanted into the right atrial septum.

#### 2.2.2 BiVP

Left ventricular lead was implanted through CS approach. BiVP was successful if the left ventricular lead was implanted in the left ventricular posterior lateral vein, and the parameters were satisfactory, the lead was fixed firmly, and there was no phrenic nerve stimulation. Otherwise change to LBBP. Atrial leads were implanted in the right atrial septum (3830), or right atrial appendage (5076).

#### 2.2.3 Programmed pacemaker

In patients without AV conduction, AV delay was set to achieve fusion with the intrinsic RV activation aiming for the shortest QRS duration. If right bundle branch could not pass down, the VV interval was adjusted and the QRS wave was narrowest through left and right ventricular pacing fusion, with right ventricular pacing (RVP) + LBBP in the LBBP group and RVP + LVP in the BiVP group.

### 2.3 Data collection and follow-up

12-lead electrocardiogram and intracardiac electrogram were recorded by GE CardioLab electrophysiological recording system. Baseline QRS duration, paced QRS duration, Stim-LVAT and procedural duration were measured. The echocardiographic data included LVEDD and LVEF (Simpson method). Lead parameters (pacing threshold, lead impedance), echocardiogram, adverse events (all-cause death, HFH, pericardial effusion, pneumothorax, Lead dislodgement, and devices infection) were collected at 1 month, 3 months, 6 months and once a year after surgery.

### 2.4 Statistical processing

Continuous variables of normal distribution are expressed by mean ± SD, continuous variables of non-normal distribution are expressed by median (interquartile distance), and categorical variables are expressed by proportion (absolute number and percentage). The UNIVARIATE method tests data normality. Univariate comparisons were performed using the t test for normal distributions and the Wilcoxon rank sum test for non-normal distributions. Chi-square test and Fisher exact test were used to compare categorical variables. SPSS 26.0 software was used for statistical analysis, and *P* < 0.05 was considered significant.

## 3 Results

### 3.1 Baseline characteristics

A total of 120 patients with HF who successfully underwent CRT-D/CRT-P were included in this study. The median follow-up time for the entire cohort was 37 ± 19 months. The mean age was 64.1 ± 9.3 years (47∼81 years), 63 patients (52.5%) were male, 120 patients (100%) were CLBBB, and 108 patients (90.0%) were diagnosed with NICM. During the operation, 3 cases tried LBBP first and changed to BiVP after failure, 4 cases tried BiVP first and changed to LBBP after failure, and finally 60 cases successfully underwent LBBP and 60 cases successfully underwent BiVP. There were no statistical differences between the two groups in demographics, medical history, baseline QRS duration, NT-proBNP, echocardiographic results and drug therapy. Table 1 describes detailed baseline characteristics for both groups of patients.

**Table 1:** Baseline characteristics.

	LBBP (*N* = 60)	BiVP (*N* = 60)	*P* value
Age, year	64.9 ± 9.1	63.2 ± 9.5	0.32
Male	35 (58.3)	28 (46.7)	0.20
NICM	54 (90.0)	54 (90.0)	1.0
HT	33 (55.0)	32 (53.3)	0.86
DM	17 (28.3)	13 (21.7)	0.40
CKD	7 (11.7)	5 (8.3)	0.54
pAF	5 (8.3)	6 (10.0)	0.75
CAD	17 (28.3)	18 (30.0)	0.84
LVEDD, mm	67.5 ± 8.9	68.9 ± 8.7	0.38
LVEF, %	29.2 ± 4.7	27.7 ± 4.4	0.08
NT-proBNP, pg/ml	3,096.3 ± 2,944.3	3,971.8 ± 3,372.0	0.13
Baseline QRSd, ms	174.7 ± 15.0	175.3 ± 14.9	0.83
Medications			
Beta-blockers	57 (95.0)	56 (93.3)	0.69
ACEI/ARB/ARNI	51 (85.0)	54 (90.0)	0.41
Aldosterone antagonist	54 (90.0)	53(88.3)	0.77

Data are shown as mean ± SD or absolute number and percentage (n%). NICM, nonischemic cardiomyopathy; HT, hypertension; DM, diabetes mellitus; CKD, chronic kidney disease; pAF, paroxysmal atrial fibrillation; CAD, coronary artery disease; LVEDD, left ventricular end-diastolic diameter; LVEF, ejection fraction; NT-proBNP, n-terminal pro-brain natriuretic peptide; ACEI, angiotensin-converting enzyme inhibitor; ARB, angiotensin receptor blockade; ARNI, angiotensin receptor neprilysin inhibitor.

### 3.2 Operation procedure

**Figure 1:**
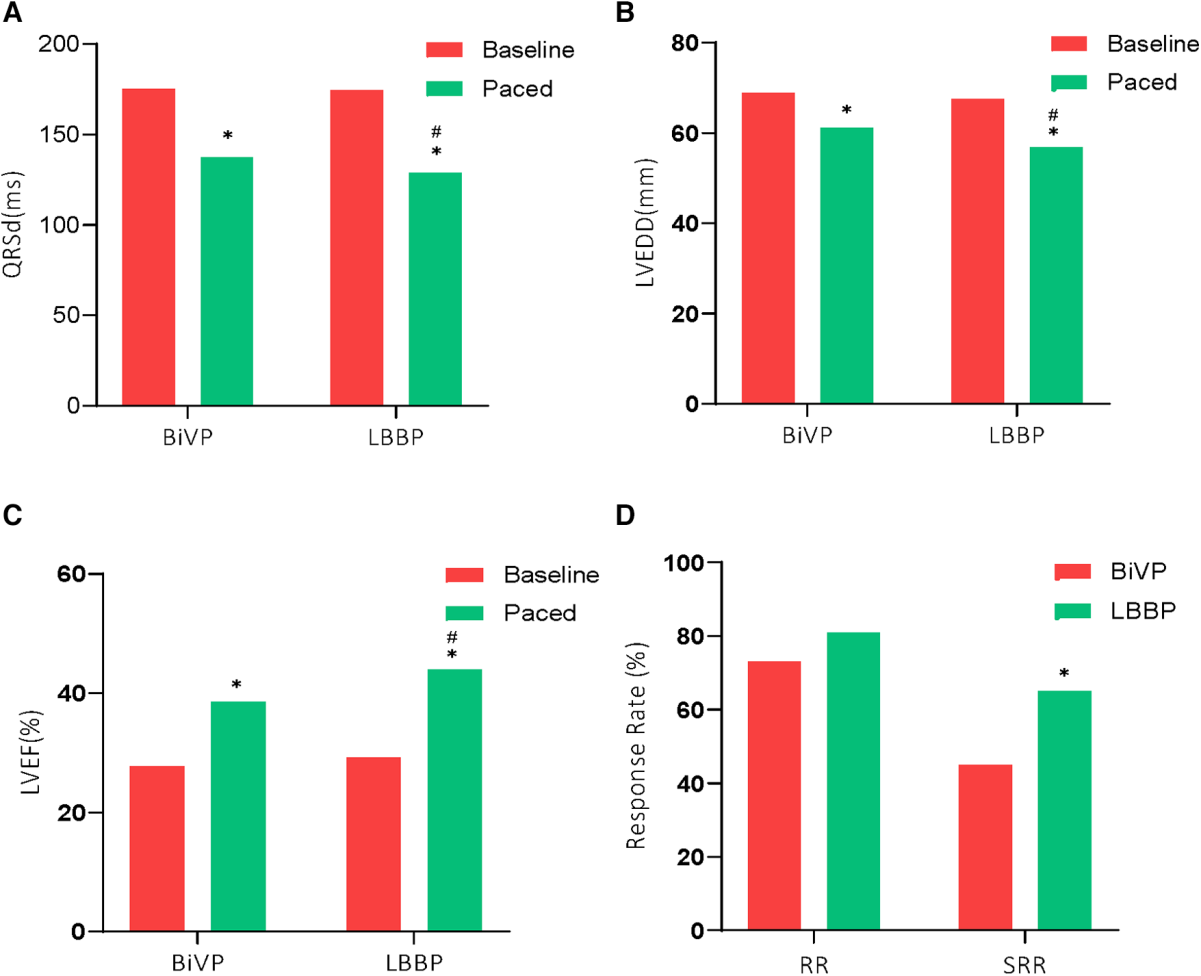
Comparison of electrical and echocardiographic results between the two groups. *P < 0.01 compared to baseline; #*P *< 0.05 compared to BiVP. Response rate (RR), and Super response rate (SRR) rates were greater with LBBP compared with BiVP.

A total of 91 patients (75.8%) received an implantable CRT-D and 29 patients (24.2%) received a CRT-P (Table 2). Compared with the LBBP group, the operation time was longer in the BiVP group (134.1 ± 32.2 min vs. 147.7 ± 39.4 min, *P *= 0.04). The LBBP group has a shorter fluoroscopy time than the BiVP group (13.7 ± 7.3 min vs. 19.3 ± 10.4 min, *P *<* *0.001). The average time of Stim-LVAT in LBBP group was 79.2 ± 9.7 min. Paced QRS duration in both groups was shorter than baseline QRS duration, and the paced QRS duration in LBBP group was more significantly shorter than that in BiVP group (128.7 ± 14.1 ms and 137.5 ± 16.5 ms, respectively (*P *= 0.002) (see Figure 1A).

**Table 2:** Results of electrical and echocardiographic and adverse events.

	LBBP (*N* = 60)	BiVP (*N* = 60)	*P* value
Procedural time, min	134.1 ± 32.2	147.7 ± 39.4	0.04
Fluoroscopy time, min	13.7 ± 7.3	19.3 ± 10.4	<0.001
Stim-LVAT, ms	79.2 ± 9.7	/	
Paced QRSd, ms	128.7 ± 14.1	137.5 ± 16.5	0.002
ΔQRSD, ms	46.0 ± 14.3	37.8 ± 14.7	0.003
NT-proBNP, pg/ml	1,364.6 ± 1,460.2	1,650.9 ± 1,441.8	0.28
Type of device			
CRT-D	46 (76.7)	45 (75)	0.83
CRT-P	14 (23.3)	15 (25)	0.83
CRT response			
Response rate	49 (81.7)	44 (73.3)	0.27
Super response rate	39 (65.0)	27 (45.0)	0.02
All-cause mortality	1 (1.7)	6 (10.0)	0.11
HFH	4 (6.7)	8 (13.3)	0.22
Complications			
Pericardial effusion	0	0	1.00
Pneumothorax	0	1	1.00
Lead dislodgement	1	1	1.00
Infection	0	0	1.00

### 3.3 Clinical results

A total of 7 patients died during follow-up, more in the BiVP group than in the LBBP group, respectively, 6 cases (10%) and 1 case (1.7%). But the difference was not statistically significant. In addition, one patient in the BiVP group still had repeated HF after operation. She received heart transplantation in the second year after operation, and the current condition is good. Compared with LBBP, there were more patients with HFH in the BiVP group, 4 and 8 cases, respectively, but the difference was not statistically significant. Postoperative echocardiographic follow-up results were obtained for all patients, as shown in Table 3. The LVEDD of both groups decreased significantly compared with that before operation (LBBP: from 67.5 mm ± 8.8 mm to 56.9 mm ± 10.9 mm; *P *< 0.001; BiVP: from 68.9 mm ± 8.6 mm to 61.1 mm ± 10.8 mm; *P *< 0.001) (see Figure 1B). The LVEDD decreased absolute value(*Δ*LVEDD) of LBBP group more significantly than that of BiVP group, which was 10.6 mm ± 8.9 mm and 7.9 mm ± 8.2 mm, respectively (*P *= 0.08). The LVEF values in both groups were improved after operation, and LBBP group improved more than BiVP group, from 29.2% ± 4.6% to 44.0% ± 9.7% (*P* < 0.001), which ranged from 27.7% ± 4.4% to 38.5% ± 9.1% (*P* < 0.001) (Figure 1C). The LVEF increase absolute value (ΔLVEF value) in LBBP group was higher than that in BiVP group (14.8% ± 9.9% vs. 10.7% ± 9.0%; *P *= 0.02). The echocardiographic response rates (ΔLVEF ≥ 5%) of LBBP group was higher than that of BiVP group, 81.7% and 73.3%, respectively (*P *= 0.27), and super response rate of LBBP group (ΔLVEF ≥ 15% or LVEF ≥ 45%) was more significant than that of BiVP group (65% vs. 45%; *P *= 0.02) (Figure 1D). In both LBBP group and BiVP group, the threshold value decreased about 1 week after operation, and then remained stable, and the threshold value rose slowly half a year later. The pacing threshold of LBBP leads were lower than that of CS leads during operation and after operation. After the acute phase, lead impedance decreased significantly and then remained stable (see Table 4). In the BiVP group, 4 patients had elevated pacing thresholds, all after 4.5 years. In acute stage, pneumothorax occurred in 1 patient in BiVP group, which was self-absorbed. During follow-up, 1 patient in the LBBP group showed that paced QRS duration widened significantly by 183 ms on the electrocardiogram after 2 years. Considering lead displacement, the original 3830 lead was removed and LBBP was performed again with new 3830 lead. Lead dislodgement occurred in 1 patient in the BiVP group, which occurred 1 month after operation. The pacemaker program control indicated that CS lead impedance was >3,000 Ω, the Lead dislodgement was found in the chest radiograph, and the CS lead position was readjusted with good follow-up parameters.

**Table 3:** Echocardiographic results.

	LBBP	BiVP	*P* value
Baseline LVEDD	67.5 ± 8.8	68.9 ± 8.6	0.37
Postprocedural LVEDD	56.9 ± 10.9	61.1 ± 10.8	0.03
ΔLVEDD	10.6 ± 8.9	7.9 ± 8.2	0.08
Baseline LVEF	29.2 ± 4.6	27.7 ± 4.4	0.08
Postprocedural LVEF	44.0 ± 9.7	38.5 ± 9.1	0.002
ΔLVEF	14.8 ± 9.9	10.7 ± 9.0	0.02

**Table 4:** Left ventricular lead parameter.

	*N*	Threshold/(V/0.4 ms)		Lead impedance (Ω)	
		LBBP	BiVP	LBBP	BiVP
Baseline	120	0.87 ± 0.50	1.47 ± 0.73^a^	736 ± 119	816 ± 251^a^
1 week	120	0.63 ± 0.31	1.23 ± 0.68^a^	462 ± 108	504 ± 158^a^
1 month	118	0.64 ± 0.17	1.11 ± 0.58^a^	453 ± 97	566 ± 147^a^
3 month	115	0.68 ± 0.26	1.20 ± 0.63^a^	431 ± 92	557 ± 140^a^
6 month	114	0.69 ± 0.17	1.22 ± 0.49^a^	416 ± 87	548 ± 137^a^
12 month	119	0.71 ± 0.22	1.23 ± 0.54^a^	403 ± 83	551 ± 152^a^
24 month	117	0.72 ± 0.21	1.39 ± 0.51^a^	389 ± 74	542 ± 140^a^

^a^ Compared with BiVP in each time period, there were statistically significant differences in threshold and impedance of LBBP.

## 4 Discussion

The results showed that: (1) ΔLVEF value in LBBP group increased significantly compared with BiVP group; (2) Compared with BiVP, ΔLVEDD decreased more significantly in LBBP group; (3) The echocardiographic super response rate of LBBP group was higher than that of BiVP group; (4) There was no significant difference between LBBP and BiVP group in all-cause mortality and HFH; (5) Compared with BiVP group, the paced QRS duration in LBBP group was narrower and the electrical synchronization was better; (6) The pacemaker threshold of LBBP group was lower than that of BiVP group, and the lead parameters of both groups were stable in the near and medium term.

BiVP-CRT has been shown in randomized large-scale clinical studies to reduce hard endpoint events such as all-cause mortality and HFH in patients with HF who have failed to respond to optimal drug therapy, LBBB, QRS wave broadening, LVEF ≤ 35%, and (NYHA) II–IV functional class. However, about 30% of patients were unresponsive to CRT, which was basically consistent with the data in this study (26.7%). The location of left ventricular lead pacing, left ventricular myocardial scar, and elevated threshold affect the CRT response rate. In addition, abnormal CS anatomy, phrenic nerve stimulation, lead dislocation and other reasons may lead to operation failure. For patients with LBBB, BiVP theoretically activates the last ventricular muscle to depolarize the left ventricle ahead of time, thereby shortening the QRS wave duration, but in fact, it does not restore the conductivity of the left bundle branch, the depolarization and repolarization sequence is not corrected, and the left ventricle still has asynchronous contraction, so it is non-physiological pacing. In 2005, HBP was first applied to a patient with HF combined with complete AVB and LBBB (7). Crossing the blocked area, the paced QRS duration wave narrowed, and the cardiac function and echocardiographic results improved significantly at 6 months of follow-up. Subsequent studies have shown the effectiveness of HBP in patients with HF combined with LBBB (8–12). HBP, as the most physiological pacing mode, can cross the proximal block area of the left bundle branch (LBB) to better achieve cardiac resynchronization and improve cardiac function. However, it can not correct all LBBBS, and it is difficult to cross the distal block area of LBB, and the success rate of correction is reported to be 75.6%∼97%. Due to the difficulty of implantation technology, high threshold, low perception, early battery exhaustion and other problems, its development is limited. In 2017, Huang et al. reported for the first time a case of LBBB patients with HF who failed to receive conventional left ventricular lead implantation with LBBP treatment, and the postoperative cardiac function improved significantly. Since then, several studies have confirmed that LBBP has become an effective physiologic pacing mode, which can achieve left ventricular mechanical synchronization similar to HBP. LBB is widely distributed in a fan-shaped network in the left ventricular septum subendocardial area and is wrapped by a small number of fibers. Its anatomical structure determines that LBBP has the advantages of simple operation, easier to cross the block area, low threshold and high perception than HBP (13,14). In this study, the success rate of LBBP surgery was 95%, which was similar to the results of other studies (15). Among them, the thickness of the interventricular septum and the insufficient supporting force of the sheath made it difficult for the 3830 lead to rotate to the left interventricular septum surface were the main reasons for the unsuccessful operation. It is expected that surgical tools for LBBP can be developed in the later stage to reduce the learning curve and further improve the success rate of operation.

A prospective multicenter study enrolled 371 patients with ischemic cardiomyopathy(ICM) or NICM receiving CRT (LBBAP or BVP) with an average follow-up of 340 days (16). The LBBAP group had 39.3% fewer HFH than the BVP group (22.6% vs. 39.5%, HR = 0.607, *P *= 0.02), but the difference in all-cause mortality was not statistically significant. A recent multiccenter observational study of 1,778 patients with CRT indicators compared the outcomes of LBBAP and BVP in the real clinical world, and showed significantly lower mortality, HF mortality, and HFH in the former with a follow-up of (33.2 ± 15.5) months (17). In the results of this study, the all-cause mortality and HFH of HF in the LBBP group were lower than those in the BiVP group, but the difference was not statistically significant, which was considered to be related to the small number of included cases. In addition, in the above study, the LBBAP group had better echocardiographic response rate than the BVP group, especially in LBBB patients, whose ΔLVEF was higher in the LBBAP group than in the BVP group (15.3%* *±* *12.0% vs. 10.8%* *±* *12.0%; *P *< 0.001). In this study, LVEF values in both groups were significantly higher than before operation, and LBBP group had a higher improvement than BiVP group (14.8%* *±* *9.9% vs. 10.7%* *±* *9.0%; *P *< 0.001), the results of echocardiographic response rate were similar to the above study. In a prospective observational study of 41 patients with LBBB, LVEF ≤ 35% and HF treated with CRT, the LBBP group showed greater improvement in LVEF (ΔLVEF) than the BiVP group after 6 months (20.5%* *±* *9.6% vs. 15.4%* *±* *11.2%, *P *= 0.15). The mean LVEF of the former was close to normal (50.9%* *±* *10.7% vs. 44.4%* *±* *13.3%, *P *= 0.12) (18). Wang et al. (6) conducted a small-scale randomized controlled study on LBBP and BiVP, including 40 HF (LVEF ≤ 40%) patients with SR, NICM and LBBB. After 6 months, LVEF improvement (ΔLVEF) in LBBP group was significantly higher than that in BiVP group (21.08%* *±* *1.91% vs. 15.62%* *±* *1.94%, *P *= 0.039). It should be noted that the improvement of LVEF in the latter two studies was significantly higher than that in this study and the previous study, which may be related to the fact that the enrolled LBBB patients all met Strauss's criteria. This suggests that echocardiographic improvement is better in patients with LBBB who meet Strauss's criteria. In Vijayaraman et al. (17) study, the echocardiographic response rate of LBBB subgroup was higher, the CRT response rate of LBBAP group was better than that of BVP group, and the echocardiographic response rate (ΔLVEF ≥ 5%) was 81.7% and 68.2%, respectively. CRT overreaction rates (ΔLVEF ≥ 20% or LVEF ≥ 50%) were 42.1% and 28.5%, respectively. In this study, the LBBP group had a higher echocardiographic response rate than the BiVP group (ΔLVEF ≥ 5%), 81.7% and 73.3% (*P *= 0.27), respectively, and the echocardiographic super response rate (ΔLVEF ≥ 15% or LVEF ≥ 45%) in LBBP patients was significantly higher than that in the BiVP group (65% vs. 45%; *P *< 0.001). Different studies have different definitions of echocardiographic response rate and super response rate, and the definition of super response rate in this study was slightly looser than that in Vijayaraman et al., so the proportion of super response rate was higher. Recently, a network meta-analysis involving 4,386 patients in 33 studies compared the clinical efficacy, electrical and lead parameters of four different pacing techniques (LBBP vs. HBP vs. BVP vs. RVP). Compared with other pacing techniques, LBBP significantly improved LVEF, shortened QRS duration, reduced HFH, had a low and stable pacing threshold, good perception, and no increased lead adverse events (19).

At present, more and more studies have proved that in patients with CRT indications, especially those NICM with CLBBB, LBBP has better efficacy than BiVP, and can be used as the first-line or preferred implantation method.

There were many shortcomings in this study: (1) Single-center, retrospective, non-randomized study; (2) The number of samples was small. Therefore, the conclusions of the study may be insufficient and need to be verified by large-scale, multi-center randomized controlled clinical trials.

## Data Availability

The raw data supporting the conclusions of this article will be made available by the authors, without undue reservation.
